# Optimizing Navigation and Text Messaging Interventions to Promote Participation in a Food Is Medicine Program Among People Participating in Cardiac Rehabilitation: Human-Centered Design Study

**DOI:** 10.2196/85650

**Published:** 2026-04-24

**Authors:** Anuroop Nirula, Rae Denise Oanesa, Leslie Castaneda, Erika Tribett, Alexis L Beatty

**Affiliations:** 1 School of Medicine, University of California, San Francisco, San Francisco, CA, United States; 2 Department of Epidemiology and Biostatistics, University of California, San Francisco, Box 0560, 550 16th Street, San Francisco, CA, 94143, United States, 1 415-562-4884, 1 415-514-8150; 3 Department of Medicine, University of California, San Francisco, San Francisco, CA, United States; 4 Project Open Hand, San Francisco, CA, United States

**Keywords:** Food Is Medicine, human-centered design, cardiovascular disease, nutrition intervention, health equity

## Abstract

**Background:**

Food Is Medicine (FIM) programs integrate interventions such as medically tailored meals or produce prescriptions into clinical care. However, there is limited evidence on how to design these programs to be responsive to the lived experiences of participants to optimize initiation, engagement, and long-term retention.

**Objective:**

The objective of the study was to develop interventions to promote initiation, engagement, and retention in FIM programs that are responsive to the lived experiences of participants.

**Methods:**

We used a human-centered design approach to engage current and former cardiac rehabilitation participants in the development of interventions to promote participation and engagement in a FIM program. We recruited participants through invitations sent via electronic health record messages. We interviewed participants about their experiences, preferences, and unmet needs related to healthy eating and program design. Additionally, we elicited participant feedback on draft versions of patient navigator scripts and text messages promoting healthy eating habits.

**Results:**

A total of six participants identified themes across Theory of Planned Behavior constructs and emergent themes, including the cost of healthy food, cultural appropriateness, clear and timely communication, transportation, local food access, scheduling flexibility, the ability to provide feedback to the program, and personalized support for navigating food resources. Participants described financial strain as a key barrier to healthy eating and noted that social influence often shaped eating behaviors. Feedback on navigator scripts led to revisions clarifying program logistics, addressing barriers such as language and cultural dietary restrictions, and tailoring positive endorsements to individual health goals. Based on participant feedback, text messages were made more concise, reframed positively (eg, humor and gratitude), and encouraged to be warmer, with respectful language that is easy to understand, while avoiding stigmatizing or overly clinical phrasing. Participants also suggested that messages should reflect empathy and offer actionable information to increase trust and engagement with the program. Trust in the health care system and a sense of dignity in receiving food support emerged as critical themes influencing overall satisfaction and retention. Participants emphasized that endorsement from their health care team and cardiologist was important for building trust in the program. Communication between health care navigators and FIM navigators could help reduce the burden placed on patients to navigate food resources.

**Conclusions:**

Using a human-centered design approach, we gained insights about participant-identified needs for navigation scripts and text messages that are culturally sensitive and personalized to promote optimal participation in a FIM program.

## Introduction

Healthy eating prevents cardiovascular (CV) events [1-4]. However, many individuals with CV conditions lack the time or resources to maintain a healthy diet. Food Is Medicine (FIM) programs can help address barriers to healthy eating. FIM interventions include providing medically tailored meals (MTMs), medically tailored groceries (MTGs), and nutritious food referrals for individuals with specific medical conditions [2,5]. These interventions can improve quality of life, improve CV risk factors, reduce hospitalizations, and reduce health care costs [2,5-9]. More specifically, MTMs improve health outcomes for those with CV conditions, especially those with barriers to planning, shopping for, or preparing healthy food. Studies of MTMs show improvements in health-related quality of life, diet quality, self-efficacy, food security, and CV risk factors (eg, blood pressure and cholesterol) [7,10,11]. MTG and healthy food referrals help support individuals who can prepare their own meals and can increase diet quality and reduce food insecurity [9,12].

Despite the benefits of FIM programs, questions remain about optimal approaches for referral, initiation, engagement, and retention in FIM programs to improve participation, engagement, and, ultimately, health outcomes [2]. Cardiac rehabilitation (CR) is a 12-week multidisciplinary program for people with heart conditions and includes counseling on nutrition and establishing long-term health habits [13,14]. CR patients are actively engaging in behavior change to prevent recurrence of future CV events; therefore, their participation in CR represents an opportune time to connect patients with FIM programs [15]. However, the best strategies for promoting the enrollment of CR patients in FIM programs are not known.

Our objective was to develop interventions to promote initiation, engagement, and retention in FIM programs that are responsive to the lived experiences of participants. We conducted human-centered design (HCD) sessions with CR patients to design 2 behavioral science–based support interventions to promote participation in a FIM program: navigation and text messaging. Navigation was adapted from approaches known for enhancing enrollment and participation in CR [16]. Text messages were based on behavioral science principles to promote initiation and engagement and were adapted from previously studied text message libraries [17,18]. These interventions are now being tested in a randomized trial for comparing the efficacy of navigation and text messaging interventions for increasing participation in FIM programs (NCT06500962).

## Methods

### Study Design

We conducted HCD sessions with individuals enrolled in CR at the University of California, San Francisco. During these HCD sessions, we collected qualitative data from participants to develop and iteratively refine the interventions through 4 phases: discover, define, develop, and deliver [19-21]. Our use of HCD is consistent with the broad principles of ISO 9241‐210, which defines HCD as an approach that centers users’ needs and requirements throughout an iterative design process. Specifically, our approach satisfied core HCD principles, including explicit understanding of users, tasks, and context (Session 1); active user involvement throughout the design process; and iterative refinement of interventions based on user-centered feedback (Sessions 2‐3). This structured, user-centered approach helps ensure that interventions are systematically refined, grounded in the lived experiences of patients, and ultimately more likely to be effective in practice. This checklist can be found in Checklist 1.

### Recruitment

We recruited a convenience sample of participants who were at least 18 years of age and participating in CR at University of California, San Francisco, an urban academic medical center. Invitations were sent via electronic health record messages. Participants were asked to provide information on gender, race or ethnicity, age, health literacy [22], and perceived social status [23]. Quantitative data were collected and managed using REDCap (Research Electronic Data Capture) electronic data capture tools.

### HCD Sessions

HCD sessions were conducted by trained facilitators over Zoom, with each session lasting 90 to 120 minutes. For each session, we held 2 groups with 2 to 4 participants each. Sessions took place between April 2024 and June 2024.

In Session 1, our aim was to understand participants’ perceptions, thoughts, and behaviors about FIM in the context of the Theory of Planned Behavior [24]. We used a template that integrated themes from the Theory of Planned Behavior to do so. This template can be found in Multimedia Appendix 1. We then took participants through a “User Journey” depicting the steps that a patient would take to participate in a FIM program called Project Open Hand. This instrument can be found in Multimedia Appendix 2. As we went through each step, we asked participants for feedback about what they would like to hear and see at each step of the journey. Based on participant responses, we developed our navigation scripts and text message library. These interventions can be found in Multimedia Appendix 3 and Multimedia Appendix 4, respectively.

Session 2 reviewed the navigation scripts and text message library with participants. Participants provided feedback on each section of the navigation script, as well as the content, length, grammar, and language of the text messages. These materials were revised based on participant feedback.

Session 3 focused on reviewing the refined navigation scripts and text messaging libraries. Additional minor revisions were made based on participant feedback.

Each session was conducted virtually using Zoom and was hosted on 2 different dates to accommodate participant scheduling needs. One primary facilitator (RDO) led each session and interviewed participants using interview guides that were drafted prior to the start of the study. The interview guides can be found in Multimedia Appendix 5. Additional members of the research team (LC and AN) were present to observe and document participant reactions and responses and to prompt further input when appropriate. The investigators did not have prior relationships with any research participants.

### Interventions

#### Navigation

We structured navigation scripts using a similar process to what is recommended for encouraging patients to enroll in CR: (1) explain the program, (2) explain the benefits, (3) provide positive endorsement, (4) describe the process to enroll, (5) welcome questions, (6) address barriers to attendance, and (7) provide follow-up information [25]. We adapted this structured approach to develop scripts for navigators to help guide CR patients through the process of enrolling and participating in a FIM program. Before enrolling in the FIM program (ie, completing their intake call with Project Open Hand staff and scheduling their first meal service), the participant will receive navigation from a health system–based navigator. After enrolling in the FIM program, the participant will receive navigation from a FIM program–based navigator.

#### Text Messaging

We developed a library of text messages to (1) promote healthy eating, (2) encourage enrollment in a FIM program, and (3) encourage adherence to a FIM program once enrolled. Text messages were designed to address behavioral science constructs using behavior change techniques [18]. Messages were 160 characters or fewer, with some including web links to relevant American Heart Association resources.

#### Analysis

HCD sessions were audio-recorded and transcribed. Audio recordings were not returned to participants for comment or correction. We qualitatively described participant responses using a rapid analysis template based on the Theory of Planned Behavior and emergent themes [24]: (1) attitudes or beliefs about FIM (thoughts or feelings about FIM and the acceptance of statements as truths or facts), (2) social influence regarding FIM (perception that an important person supports a behavior), and (3) perceived behavioral control. The template we used to analyze participant responses can be found in Multimedia Appendix 1. Three study staff members (AN, LC, and RDO) coded responses individually to extract themes and representative quotations from each transcript before meeting to achieve consensus. Analyses were completed concurrently with data collection. We calculated descriptive statistics (eg, mean and SD) for participant demographic information using STATA version 18 (StataCorp LLC).

### Ethical Considerations

This study was approved by the WCG Institutional Review Board (1370693), and procedures were followed in accordance with WCG Institutional Review Board and institutional guidelines. Prior to the start of the first session, participants provided written informed consent. They also provided verbal consent for audio recording prior to the beginning of each session. To protect participant confidentiality and to minimize the possibility of sharing information that could be used for potential reidentification, session transcripts will not be available; however, analysis files may be obtained from the corresponding author upon reasonable request. Participants were compensated US $200 per HCD session for their time and participation.

## Results

### Overview

Twenty-two people were invited to participate in the study: 6 consented and participated, 1 consented but did not participate in any study activities after providing consent, 3 were found to be ineligible, 1 explicitly declined, and 11 did not respond. We enrolled 6 participants, of whom 4 were female and 2 were male, 2 self-identified as Black or African American and 4 as White. All participants spoke English. Participants ranged in decade of age from their 40s to their 80s. Mean perceived social status was 6 (SD 2.6; scale from 1 lowest to 10 highest). Mean health literacy was 2.8 (SD 1; scale from 0 to “Not at all confident filling out medical forms” to 4 “Extremely confident”).

### Qualitative Findings

In addition to prespecified themes from the Theory of Planned Behavior, which are (1) attitudes or beliefs about FIM, (2) social influence regarding FIM, and (3) perceived behavioral control, we also identified emergent themes: (4) cost of shopping for healthy foods, (5) culturally appropriate food, (6) local food options, (7) flexibility with FIM programs, (8) ability to give feedback to FIM programs, and (9) personalization. Qualitative themes are described in additional detail below.

#### Theme 1: Attitudes and Beliefs About FIM

There was an overall understanding among participants that eating healthy foods is important, particularly for maintaining one’s health. Participants preferred to make healthier choices and believed that FIM programs can improve eating habits and instill the confidence needed to eat and cook healthier foods.

*If I have a little more vegetables in my daily diet, I think it will make me feel a little better on the inside.*

[P5]

*This program [FIM] will help with your recovery, because the food is more based on what you need to eat.*

[P4]

*[The FIM program] is helping you to change your way of eating… to keep your heart healthy.*

[P4]

#### Theme 2: Social Influence Regarding FIM

There was a general understanding among participants that different individuals will influence eating habits in various ways, thus impacting the ability to adhere to diets recommended by FIM programs. Some of these individuals were family members, friends, and the participant’s clinical team (doctors, dietitians, etc). Regardless of who they are, participants agreed that eating habits are influenced by the people one eats with, whether negatively or positively.

##### General

*I mean when I’m at a table of six or eight people, and everybody has dessert but me? Well, I don’t want to get left out, so I’m more likely to have dessert.*

[P3]

*I think normally we have sort of a social norm of, you know, three meals a day. It’s gonna be balanced. It’s gonna be complex a little, and if you’re with other people or something like that, it’s gonna be nicer. You’re not just gonna be like, you know, okay here’s a bowl of mashed potatoes and nothing to go with them.*

[P6]

##### Family

*Some of the stuff that I would cook and prepare, that I was raised on, [my children] don’t eat, so I had to start cookin’ stuff that they would eat, so that kind of changed my eating habits. So my menu mostly revolves around what they would eat.*

[P5]

*If you’re working with someone who is cooking for others, children, whoever is in their household, if they are eating different foods from the family, it would be a family affair…. if the family’s eating habits don’t change, that would be a big barrier to maintaining change achieved through Project Open Hand.*

[P3]

##### The Clinical Team

*Somebody to just give you guidance and not wait until we have these heart conditions to do so.*

[P4]

*The only person who wants me to eat healthy are my doctor, my cardiologist, and people in the health field. And myself.*

[P4]

### Theme 3: Perceived Behavioral Control

Participants generally seemed motivated to eat healthier and agreed that they had all developed different habits unique to their current situations. While there is a consensus that individuals would like to eat healthier, the challenge often lies in having the energy and knowledge to prepare nutritious meals.

Individuals explained habits that they have developed around cooking and eating food based on their current situations and perceived ability to make certain foods.

*I’m in super bachelor mode… often I’ll just fix one item and just eat that… often that’s about as much as I can do sometimes. Since I’m not really sharing it with anyone… it is just getting food in.*

[P6]

*I love eating, but I’m also lazy… I’m not gonna get up and make, you know, a 7-vegetable omelet.*

[P1]

*Even with your friends…The bottom line comes to us. Taking control over what we put in our bodies.*

[P4]

Furthermore, participants shared that part of the barrier to eating healthy lies in shopping for healthier foods and groceries. These barriers are often out of one’s control. There are multiple factors that individuals must consider when grocery shopping, including cost, current living situation, and shelf life.

*[Finding healthy food to eat] is also the combination of like, you know, not only is this, is this good? Is this healthy? But then you know the price and when you have your budget in one eye and doing calculations in the other eye… can be stressful on its own frankly.*

[P6]

*I will make compromises, and I will hit the pasta aisle, and I will hit the ramen aisle and all of the things that last forever.*

[P6]

*I think [the senior living center] buys a hundred pounds of whatever vegetable they’re having. So we get the same vegetables. I love broccoli, but you know by the seventh day in a row I’d rather have something else*.

[P3]

### Theme 4: Cost

Participants expressed a strong desire to eat healthy but acknowledged that this lifestyle can be prohibitively expensive. They also highlighted the stress of budgeting and calculating what they can afford. As a result, many end up eating only what fits their budget, even if it is not healthy.

*Money restrictions create a lot of bad health food choices, or bad food choices, I think.*

[P6]

*I’ll be looking at things just like, oh I really want that, but I can’t afford it. So therefore I don’t get it. So I wind up getting something else. I shop a lot of sales.*

[P4]

*Often, it comes down to ‘How many pennies per calorie is this’ rather than price per ounce or gram… ‘Okay how long can I live off of this stuff?’*

[P6]

### Theme 5: Cultural Appropriateness

Cultural appropriateness refers to the idea that there is a match between the foods provided and the cultural food traditions of the patient. Participants cited that the cultural appropriateness of foods is a factor that impacts their eating habits. Therefore, it can be understood that cultural appropriateness is particularly crucial in the context of FIM programs, as it affects the patient’s ability to adhere to the meal plans or cook with the groceries provided.

*I’m a country girl, we like ham, hogs, mustard greens, collard greens, pinto beans, black-eyed peas… we like that good Southern cookin,’ but when I came up here to California, all that stuff I see back home, they don’t sell up here. So I have to make my menu list different*

[P5]

### Theme 6: Local Food Options

Many individuals expressed a strong desire to obtain fresh groceries and support local farmers’ markets, as they believe these items are inherently healthier. However, they find farmers’ markets challenging to access due to high prices.

*It’s the priciness of it, I would totally support the local farmers over anybody else.*

[P4]

*The Farmers’ Market is like goin’ out there and pickin’ it yourself. So you know it is fresher, and like I say, fresher is better.*

[P5]

### Theme 7: Flexibility of FIM Programs

Regarding FIM programs, there was widespread consensus that the program needs to be flexible (ie, with scheduling food services, switching between MTM and MTGs), as patients will have other commitments such as rehabilitation sessions or doctor’s appointments and may desire having meals or groceries delivered depending on their current situation.

*Showcasing your flexibility here and there. So many of these programs are basically like ‘Do this or you’re disqualified.’ That adds a lot of stress.*

[P6]

### Theme 8: Feedback

Individuals also emphasized that FIM programs should create space for feedback as it empowers patients to be active participants in the program and helps ensure the program remains responsive and adaptable to their needs.

*Lack of ability to express feedback may be a barrier. If after a month you’re getting bored with it, and you can’t express that boredom, you might give up.*

[P2]

*Let people know they can say “Hey I prefer this type of food over this.*

[P2]

### Theme 9: Personalization

Participants also stressed the need for personalizing the FIM program to each individual. When implementing and explaining the program, they suggested that explanations should be related back to the patient’s unique circumstances. This approach will clarify how the program will impact them personally and will likely increase their motivation and engagement.

*It will give you tools to learn how to eat appropriately for your body and health concerns.*

[P1]

*Why is this here? What is this gonna do for me?*

[P6]

These representative quotes, as well as some others, are listed below in Table 1.

**Table 1.** Representative quotes from participants regarding Theory of Planned Behavior constructs and emergent themes.

Theme	Representative quotes
Theory of Planned Behavior themes	
Attitudes and beliefs about FIM^a^	*“If I have a little more vegetables in my daily diet, I think it will make me feel a little better on the inside.”* (P5) *“This program will help with your recovery, because the food is more based on what you need to eat.”* (P4) *“[The FIM program] is helping you to change your way of eating… to keep your heart healthy.”* (P4)
Social influence regarding FIM	General *“I mean when I when I’m at a table, of six or eight people, and everybody has dessert but me? Well, I don’t wanna get left out, so I’m more likely to have dessert.”* (P3) *“I think normally, we have sort of a social norm of, you know, 3 meals a day. It’s gonna be balanced. It’s gonna be complex a little, and if you’re with other people or something like that, it’s gonna be nicer. You’re not just gonna be like, you know. Okay, here’s a bowl of mashed potatoes and nothing to go with them.”* (P6) Family *“Some of the stuff that I would cook and prepare, that I was raised on, [my children] don’t eat, so I had to start cookin’ stuff that they would eat, so that kind of changed my eating habits. So my menu mostly revolves around what they would eat.”* (P5) *“If you’re working with someone who is cooking for others, children, whoever is in their household, if they are eating different foods from the family, it would be a family affair... if the family’s eating habits don’t change, that would be a big barrier to maintaining change achieved through Project Open Hand.”* (P3) The Clinical Team *“Somebody to just give you guidance and not wait until we have these heart conditions to do so.”* (P4) *“The only person who wants me to eat healthy are my doctor, my cardiologist, and people in the health field. And myself.”* (P4)
Perceived behavioral control	General *“I’m kind of in super bachelor mode... often I’ll just fix one item and just eat that... often that’s about as much as I can do sometimes. Since I’m not really sharing it with anyone... it is just getting food in.”* (P6) *“Just getting nutrition in front of me for the most part would be preferable.”* (P6) *“Even with your friends...The bottom line comes to us. Taking control over what we put in our bodies.”* (P4) *“You have to be your own cheerleader... when it comes to your health.”* (P4) Buying Food/Groceries *“I will make compromises, and I will hit the pasta aisle, and I will hit the ramen aisle and all of the things that last forever*. (P6) *“[Finding healthy food to eat] is also the combination of like, you know, not only is this, is this good? Is this healthy? But then you know the price and when you have your budget in one eye and doing the calculations in the other eye… can be stressful on its own frankly.”* (P6) Cooking/Preparation *“I [freeze my food] because I’m afraid that I might run out of money and I’m afraid that I don’t wanna run out of food. So I try to stretch what I get.”* (P4) *“I love eating, but I’m also lazy… I’m not gonna get up and make, you know, a 7-vegetable omelet.”* (P1) Eating *“Although there will be days of scarfing pasta and mashed potatoes and things like that.”* (P6) *“I think [the senior living center] buys a hundred pounds of whatever vegetable they’re having. So we get the same vegetables. I love broccoli, but you know by the seventh day in a row I’d rather have something else.”* (P3) *“I’m a foodie person, so I’d like to make different things, and I get bored if I eat the same thing over.”* (P4)
Emergent themes	
Cost	*“Money restrictions create a lot of bad health food choices, or bad food choices, I think.”* (P6) *“I’ll be looking at things just like, oh I really want that, but I can’t afford it. So therefore I don’t get it. So I wind up getting something else. I shop a lot of sales.”* (P4) *“Often it comes down to ‘how many pennies per calorie is this’ rather than price per ounce or gram... ok how long can I live off of this stuff.”* (P6)
Cultural appropriateness	*“I‘m a country girl, we like ham, hogs, mustard greens, collard greens, pinto beans, black-eyed peas... we like that good Southern cookin,’ but when I came up here to California, all that stuff I see back home, they don’t sell up here. So I have to make my menu list different.”* (P5)
Local food options	*“It’s the priciness of it, I would totally support the local farmers over anybody else.”* (P4) *“The Farmers’ Market is like goin’ out there and pickin’ it yourself. So you know it is fresher, and like I say, fresher is better.”* (P5)
Flexibility	*“Showcasing your flexibility here and there. So many of these programs are basically like ‘Do this or you’re disqualified.’ That adds a lot of stress.”* (P6)
Feedback	*“Lack of ability to express feedback may be a barrier. If after a month you’re getting bored with it, and you can’t express that boredom, you might give up.”* (P2) *“Let people know they can say ‘Hey I prefer this type of food over this.”* (P2)
Personalization	*“It will give you tools to learn how to eat appropriately for your body and health concerns.”* (P1) *“Why is this here? What is this gonna do for me?”* (P6)

a FIM: Food Is Medicine.

### Intervention Feedback

#### Navigation Scripts

Overall, participants recommended that navigators take the time to thoroughly learn about each participant by asking in-depth and detailed questions in each section of the script. This will help personalize the intake process as much as possible.

Examples of the feedback received in response to the navigation script are listed in Table 2.

**Table 2.** Description of feedback and modifications for navigation scripts.

Section	Feedback
Explain what FIM^a^ is	Differentiate between a nutritionist and a dietitian. If the program is using a dietitian, then the word “nutritionist” should be replaced with “dietitian” as a dietitian is perceived to provide a higher level of care due to differences in training. Clarify whether the program is just for the participant, or if it includes the participant’s entire family. Instead of presumptuous statements, such as “I will also learn more about you,” use “I hope to learn more about you and how we can help you learn about eating healthier.”
Benefits	Indicate that the program is “Integrating care together” (ie, FIM works with the clinical team to provide care). *“Lay out the cause and effect... here is [the program’s] medicinal value.”*
Positive endorsement	Indicate “demonstrable effects” - *“Why would someone want this and need this—what’s it gonna do for them?”* Behavior modification: *“The food is designed for you to start looking at food differently for your heart health*.” Individually tailor reasons as to why FIM would be beneficial for the participant.
Process to enroll	It is unclear who is calling whom, make the process clearer. Indicate when the participant is talking to the health system navigator and the FIM navigator. The part that states “We will send in an enrollment form to Project Open Hand” is not clear. Who is sending the form to whom? How are you sending it? Overall, participants liked that the health system navigator is communicating with the FIM navigator. One participant remarked that it *“allows the person to take the stress out of the food situation*” and is “*part of the healing*.”
Discuss any barriers	Participants gave examples of barriers to discuss: Language Cultural or dietary restrictions Lack or loss of interest in the program (due to multiple reasons: they don’t like it, they don’t see positive results, etc) Lack of ability to give feedback to the program (creating space to allow participants to express feedback so that the program can better suit their needs)

a FIM: Food Is Medicine.

#### Text Messages

##### General Comments

We received both general suggestions and detailed edits for our text messages. Overall, participants preferred messages with positive affect (eg, gratitude, humor). They also recommended framing messages to incentivize positive behavior change and incorporating language around harm reduction rather than instructing patients to completely avoid certain foods. Concise text messages with examples of recommended foods and changes were preferred. Participants also expressed a desire for 2-way text messaging with the ability to have a conversation with a health professional such as a dietitian.

##### Length

We received mixed feedback regarding the length of text messages. Some participants recommended making messages longer to justify our recommendations, whereas others preferred the messages to be concise. To incorporate both sides of the feedback, we shortened some messages while including external links that participants could access to learn more.

##### Grammar

Participants found some text messages confusing and provided feedback regarding clarifying the language and prose.

##### Behavior Modification and Encouragement

The importance of positively reframing messages to encourage behavior change was also stressed. Furthermore, participants suggested that providing clear and concrete examples when suggesting actions can serve as an additional incentive for individuals to follow through.

##### Providing Resources

There was a consensus that any text messages recommending an action, such as enrolling or calling the FIM program, should include a phone number for contacting the FIM program. Examples of how text messages were modified in response to participant feedback are shown in Table 3.

**Table 3.** Text message modifications.

Feedback	Original	Modification
Short messages with links to learn more		
	Avoid nighttime eating! There is nothing wrong with a healthy snack after dinner, but avoid large amounts of food high in calories or fat.	Avoid nighttime eating! If you are going to eat, choose a fruit or vegetable. Click to learn more!
	Remove temptation by getting rid of all the convenient, unhealthy snacks.	Remove temptation by getting rid of all the convenient, unhealthy snacks. Click to learn what is considered an unhealthy snack.
Easy to read		
	Learning how to read food labels can help you make healthier choices. Choose lower sodium, saturated fat and added sugars.	Reading food labels can help you make healthier choices. Choose low sodium, low saturated fat, and low added sugars. Click to learn more!
	Eating whole-grain foods can help lower your cholesterol. Oatmeal, whole-grain cereals, brown rice, whole-wheat bread, and rye bread are all whole-grain foods.	Oatmeal, whole-grain cereals, and brown rice are some whole-grain foods that will help lower your cholesterol. Click to learn more!
Behavior modification and encouragement		
	Eating too fast or when you’re distracted may lead to consuming too many calories—slow down!	Savor the flavor! Eating too fast or when you’re distracted may lead to consuming too many calories. Click to learn more!
	Almost done with Project Open Hand? Don’t give up on all your healthy choices—create a plan to keep yourself going.	Almost done with Project Open Hand? Create a plan to keep yourself going—start by writing down your favorite foods from Project Open Hand.
	Feeling tired? Eating healthy foods can help give you more energy. Project Open Hand can help!	Feeling tired? Join Project Open Hand to eat heart-healthy foods and gain more energy! Call now at 415-447-2326!
Providing resources		
	Want to take a positive step toward a healthier life? Sign up for Project Open Hand today!	Want to take a positive step toward a healthier life? Sign up for Project Open Hand today by calling 415-447-2326.
	Make a plan for when you will sign up for Project Open Hand.	Make a plan and set some time aside for when you will sign up for Project Open Hand. Call now at 415-447-2326!

## Discussion

### Principal Results

Using HCD principles, we worked with people participating in CR to develop 2 interventions to promote enrollment and engagement in a FIM program: navigation and text messaging. Our findings suggest that, overall, participants held positive beliefs about the impact of FIM programs and understood the importance of having a healthy diet. Despite this understanding, there were numerous factors impacting individuals’ eating habits, such as personal beliefs about healthy eating, social influences on the eating habits, cost, and availability of healthy foods. Furthermore, participants emphasized that FIM program design features, such as flexibility, ability to offer feedback, and personalization were important for remaining engaged.

Other barriers, such as financial feasibility and access to healthier foods, were also discussed. Fresh produce is often more expensive and not as readily available locally, and this may impose a financial burden on individuals who come from low-income backgrounds [26]. This leads to unequal access to a healthier diet, further exacerbating the disproportionate burdens and impacts of cardiac illnesses in these populations [27]. Understanding healthy eating within the context of these financial strains is crucial for developing interventions that enhance the effectiveness of FIM programs.

Participants noted that their cardiologist and health care team are key figures in encouraging healthy behaviors. Thus, they emphasized that partnership between these providers and the FIM program is important—not only to monitor the FIM program’s impact on their CV health but also to improve access to these programs, since physicians can often play a central role in initiating referrals. Other studies have similarly found that a strong partnership between health care providers and food pantries, along with a consistent availability of healthy food resources, is vital in ensuring the effectiveness of food prescription programs. For example, one study employed clinic-based interventions in coordination with local food banks and showed measurable improvements in patient outcomes, such as improved blood sugar and blood pressure control [27]. Another qualitative study in Houston revealed that program success relies on clear workflows between clinicians and food pantry staff and the integration of food prescriptions into routine health care [28].

The use of HCD to engage people with lived experiences in the development of an intervention may improve the relevance and acceptability of health behavior interventions. The feedback provided by participants encouraged positive framing, the offering of examples, and practical actions rather than the presentation of information about health consequences. The feedback provided to us demonstrated similar themes consistent with findings from other studies that have used HCD sessions for text messaging. For example, in a study that focused on developing text messages for the prevention of CV disease in people with HIV, participants similarly preferred messages that were short, clear, and actionable. They emphasized the importance of a positive tone over judgmental language and valued advice that was culturally relevant [16]. These patterns, similar to those found in our study, suggest that text message–based interventions are most effective when they deliver brief, actionable, and supportive messages that align with the contextual realities of patients. This approach, if used in future text-based interventions, may enhance engagement, promote behavior change, and ultimately reduce disparities in health outcomes.

Personalizing FIM programs to align with individuals’ unique health goals and nutrition literacy is important for maximizing their effectiveness. Research shows that when nutrition interventions are tailored to an individual’s knowledge level and perceived self-efficacy in making dietary changes, they are more likely to adopt and sustain healthy behaviors [29]. This encourages individuals to make better dietary choices, leading to increased fruit and vegetable intake and a greater ability to manage decisions related to food. As a result, individualized approaches not only allow for greater access to healthier foods but also allow for the development of the skills and confidence required to use them effectively.

### Limitations

Despite having many strengths, our study has limitations that should be acknowledged. Our HCD sessions were conducted with a small number of participants, with each session consisting of only 3 to 4 participants. While a sample of 6 participants is appropriate for our study design, our sample included only participants who identified as Black or African American and White. This may limit the generalizability of our study’s findings. Future research should prioritize replicating the findings of this study with larger and more racially and ethnically diverse samples to reach data saturation and ensure that FIM program interventions are generalizable and culturally responsive across populations. Furthermore, our study was conducted at a single urban academic medical center CR program, which may also limit the generalizability of our findings for individuals with other chronic conditions and those who may benefit from FIM. Individuals who do not live in urban areas, are enrolled in other rehabilitation programs, or have other chronic conditions not related to cardiac health may offer different perspectives and feedback regarding their unique experiences with healthy eating and the impact of FIM programs. Additionally, the virtual format of our HCD sessions may have impacted the quality of discussion. While conducting the sessions over Zoom ensured that all participants could attend, it may have influenced the way in which participants engaged in discussions. Furthermore, HCD ideally incorporates direct observation of participants engaging with the intervention being developed, a method that was not feasible with the virtual format of our sessions. This may have limited our ability to capture feedback on real-world usability and real-time behavioral and nonverbal responses to our interventions. Future research should incorporate field observation to further refine these interventions. The interventions developed in our study may need to be adapted for use in other settings.

Connecting CR and FIM programs offers a scalable opportunity to improve healthy eating and health outcomes in people with heart conditions. HCD and behavioral science approaches are promising in the development and execution of effective, coordinated nutrition care for these individuals. This research will inform future studies testing the feasibility and efficacy of navigation and/or text messaging to improve participation in FIM among patients with heart disease.

## Supplementary material

10.2196/85650 Multimedia Appendix 1 Rapid analysis template.

10.2196/85650 Multimedia Appendix 2 Participant user journey.

10.2196/85650 Multimedia Appendix 3 Test message library.

10.2196/85650 Multimedia Appendix 4 Health system navigator script and Food Is Medicine navigator script.

10.2196/85650 Multimedia Appendix 5 Participant interview guides.

10.2196/85650 Checklist 1 COREQ 32-item checklist.

## Abbreviations

COREQ: Consolidated Criteria for Reporting Qualitative Studies
CR: cardiac rehabilitation
CV: cardiovascular
FIM: Food Is Medicine
HCD: human-centered design
MTGs: medically tailored groceries
MTM: medically tailored meal
REDCap: Research Electronic Data Capture
