# Supplementary material for: Optimizing Navigation and Text Messaging Interventions to Promote Participation in a Food Is Medicine Program Among People Participating in Cardiac Rehabilitation: Human-Centered Design Study
Source: JMIR Form Res. 2026 Apr 24;10:e85650. doi: 10.2196/85650 (PMC13122297; doi:10.2196/85650)
Supplement: Multimedia Appendix 1 [file formative-v10-e85650-s001.docx]

**Rapid Analysis Template**

| **Theme** | **Quotes** | **Reflections** |
| --- | --- | --- |
| **Theory of Planned Behavior** |  |  |
| **Attitudes & beliefs about FIM**  *Thoughts or feelings about FIM. Acceptance of statements as truths or facts.*  *Examples: “I know healthy food is good for me.” “I just don’t like broccoli”* |  |  |
| **Social influence re: FIM**  *Perception that an important person supports a behavior.*  *Example: “My doctor told me to eat more fruits and vegetables.”* |  |  |
| **Perceived behavioral control**  *Perception of the ability to perform a behavior.*  *Example: “I need a shopping list for healthy foods.” “I don’t know how to sign up for the program”* |  |  |
| **Other** |  |  |
| **Cost**  *Costs of the intervention and costs associated with implementing the intervention.*  *Example: “Buying healthy foods costs so much.”* |  |  |
| **Cultural appropriateness**  *A match between the foods provided and the cultural food traditions of the patient.*  *Example: “The flavors don’t match what I am used to.”* |  |  |
| **Emergent themes** |  |  |
|  |  |  |
| **General Reflections** |  |  |
| **Recommended Revisions to Materials** |  |  |
