# Supplementary figures and images for: Optimizing Navigation and Text Messaging Interventions to Promote Participation in a Food Is Medicine Program Among People Participating in Cardiac Rehabilitation: Human-Centered Design Study
Source: JMIR Form Res. 2026 Apr 24;10:e85650. doi: 10.2196/85650 (PMC13122297; doi:10.2196/85650)

**Figure S1. User Journey**


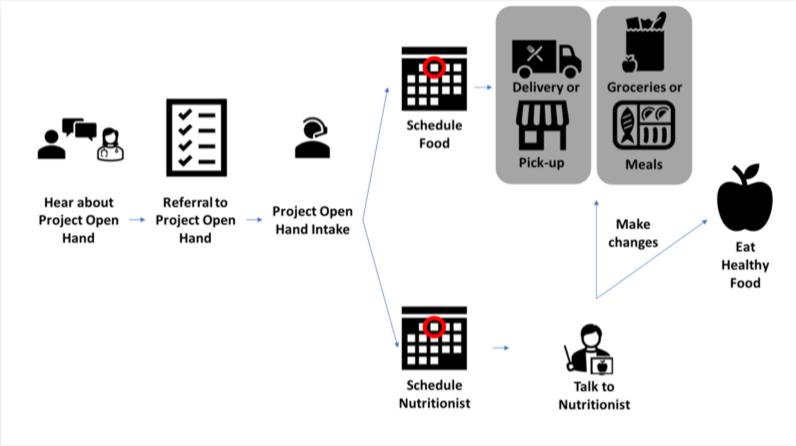


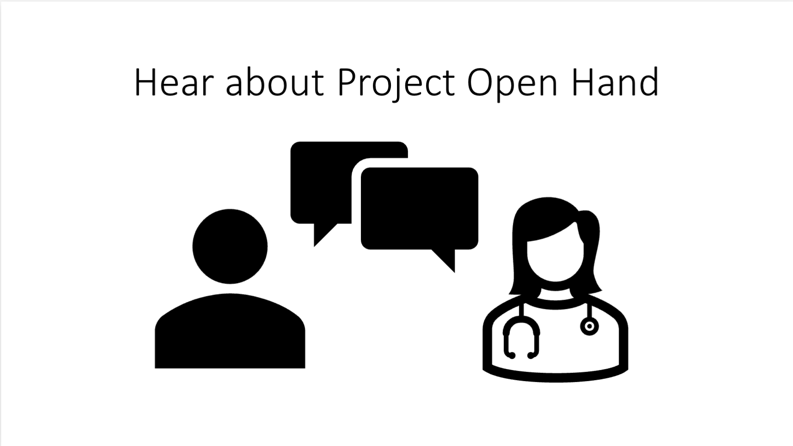

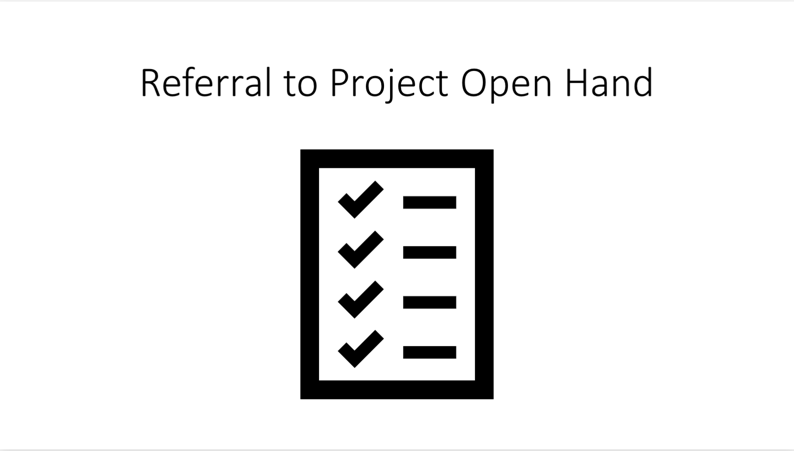


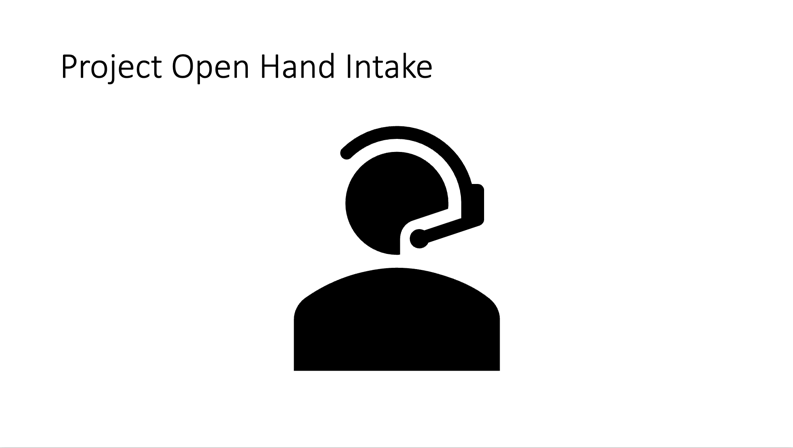


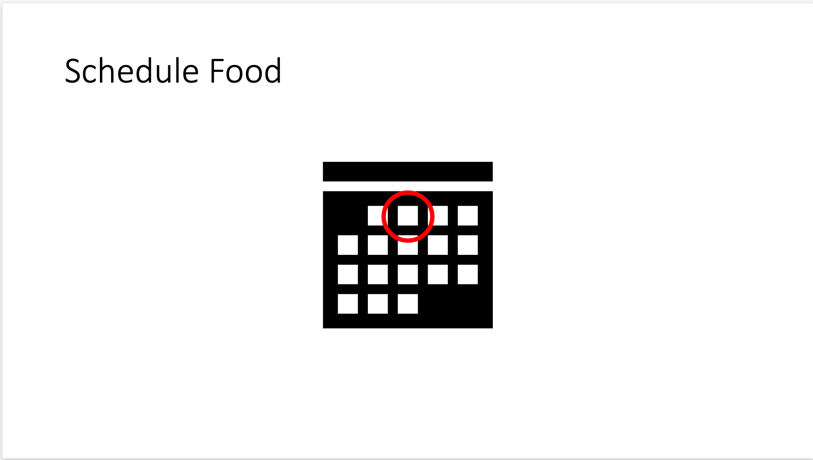


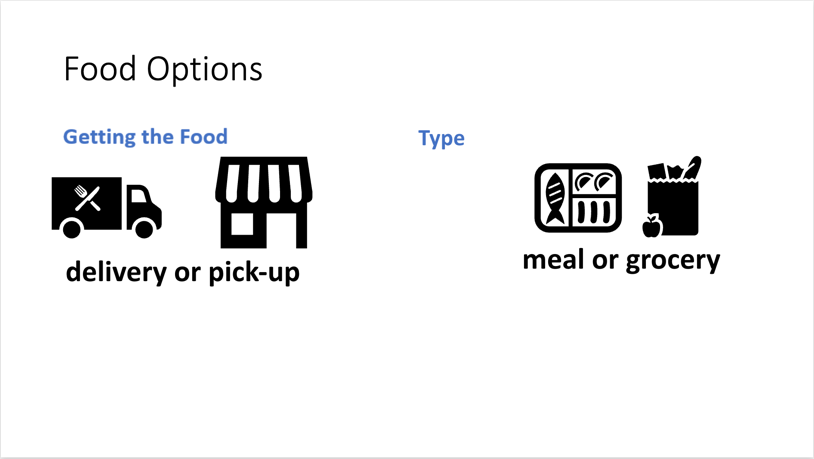


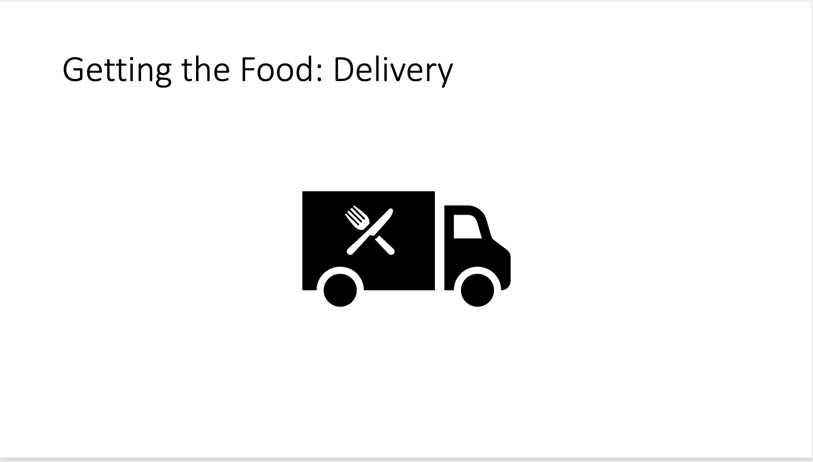


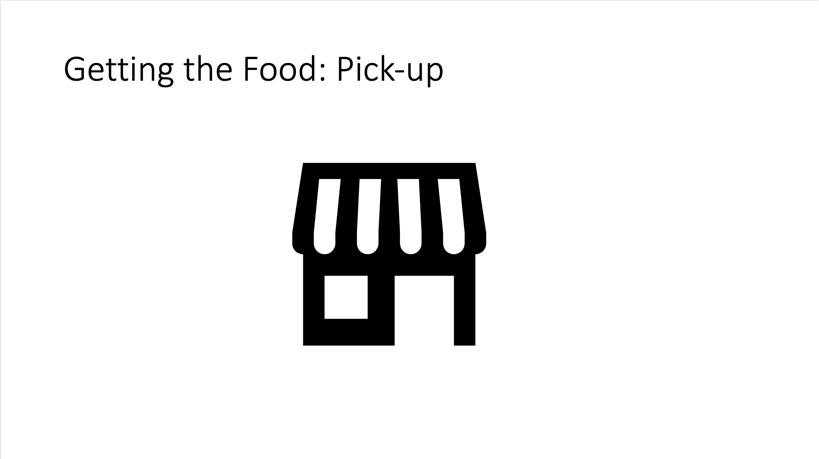


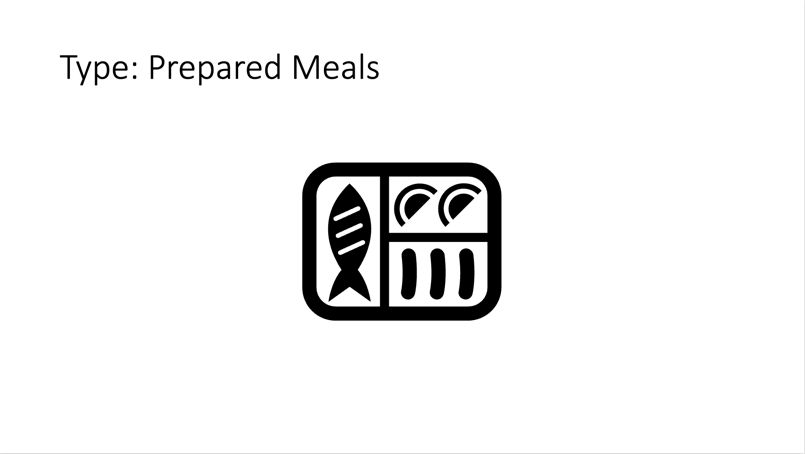


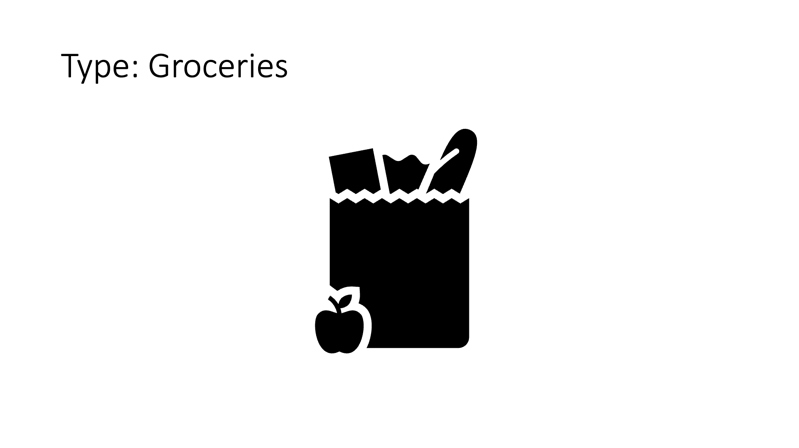


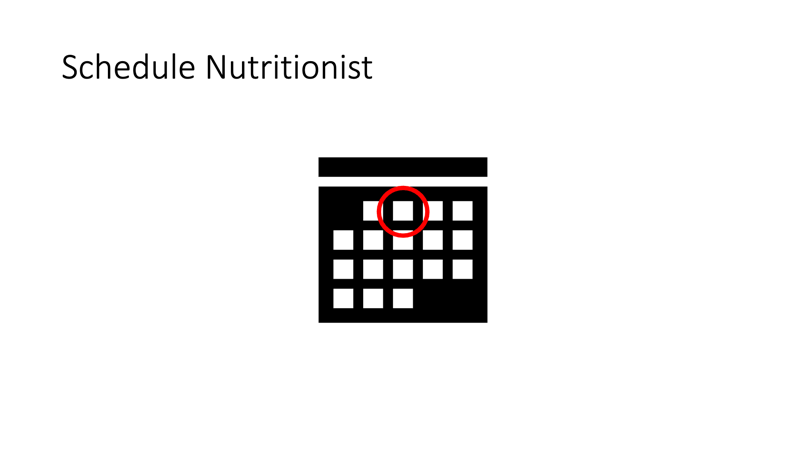


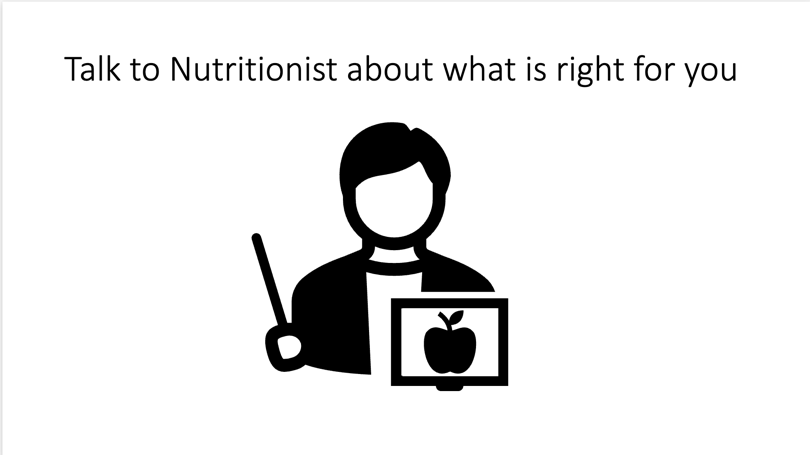


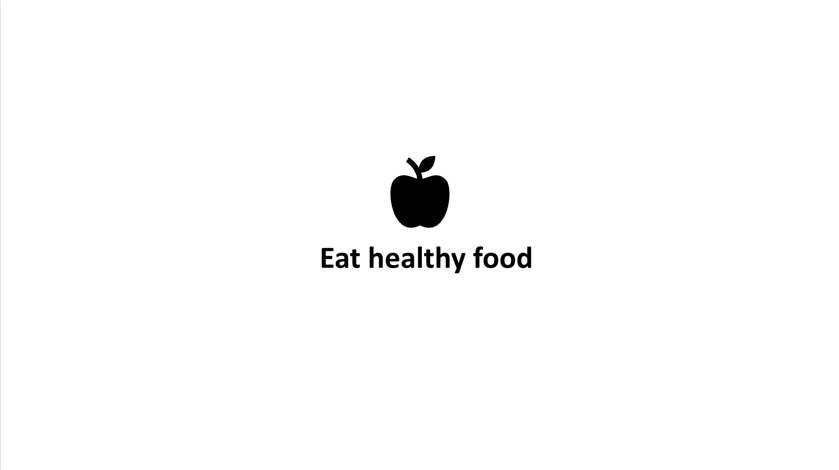


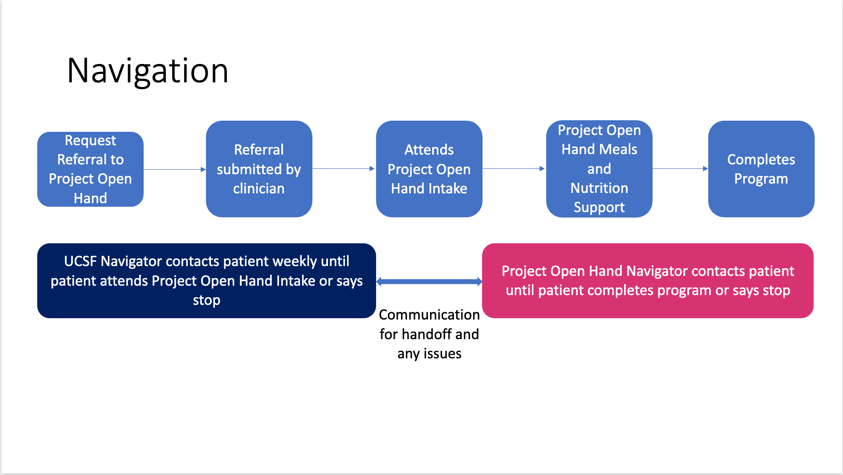


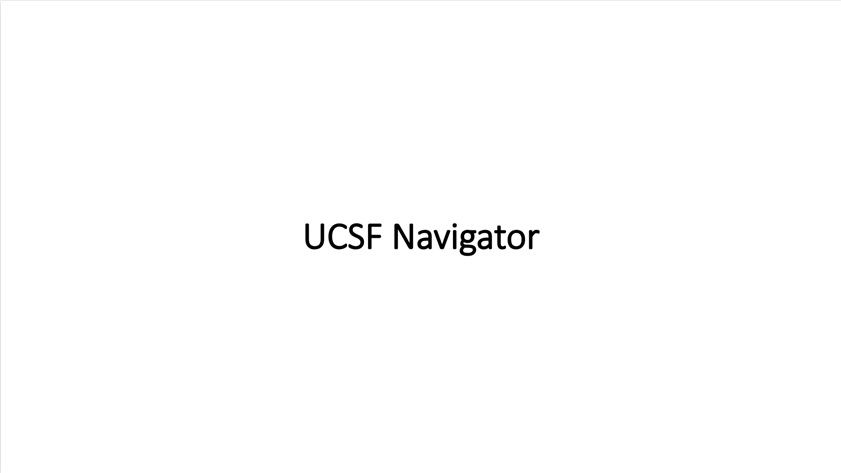


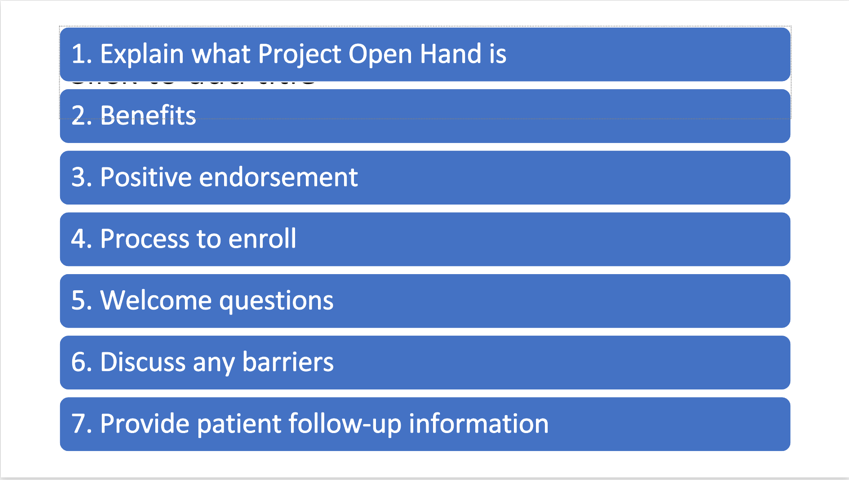


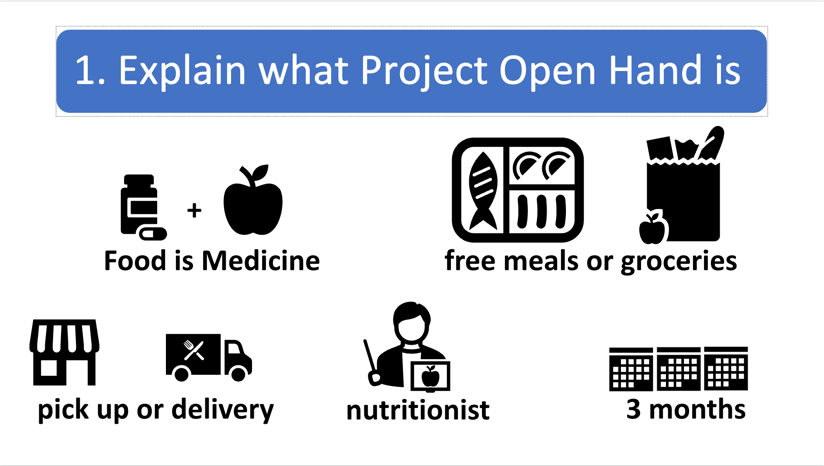


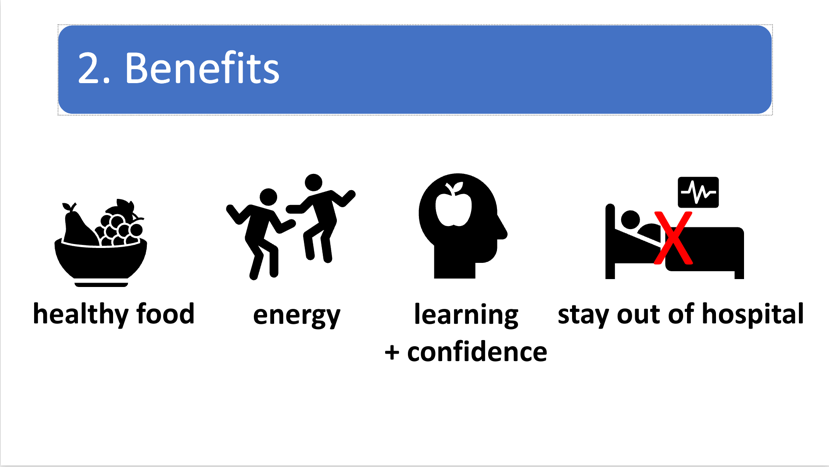


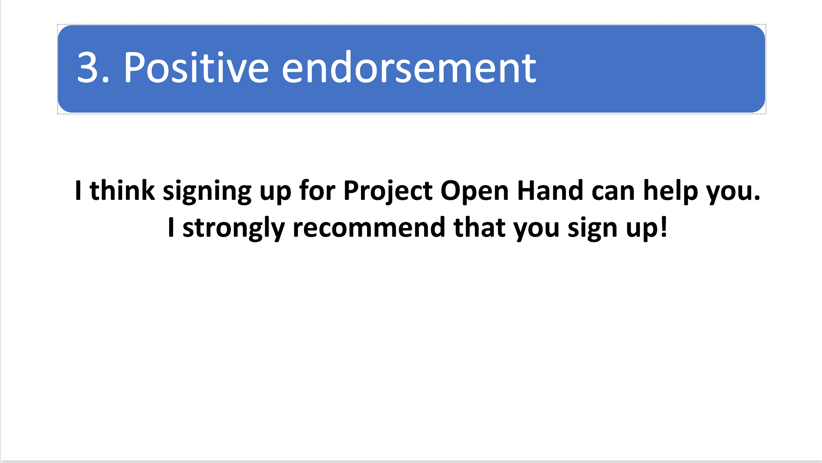


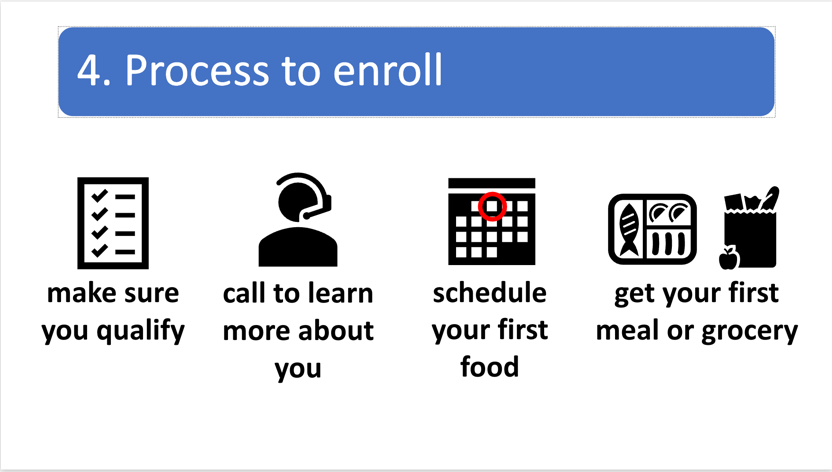


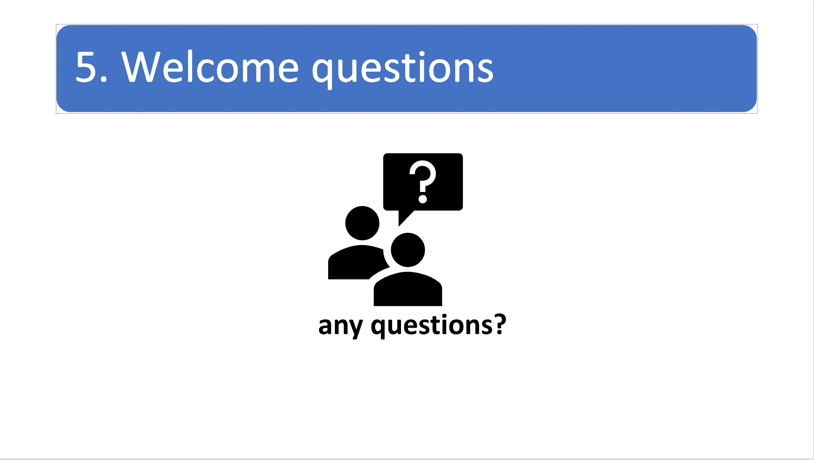


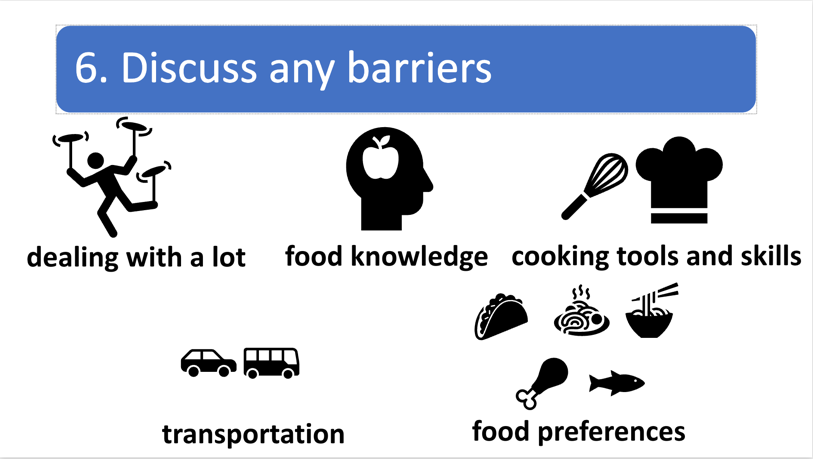


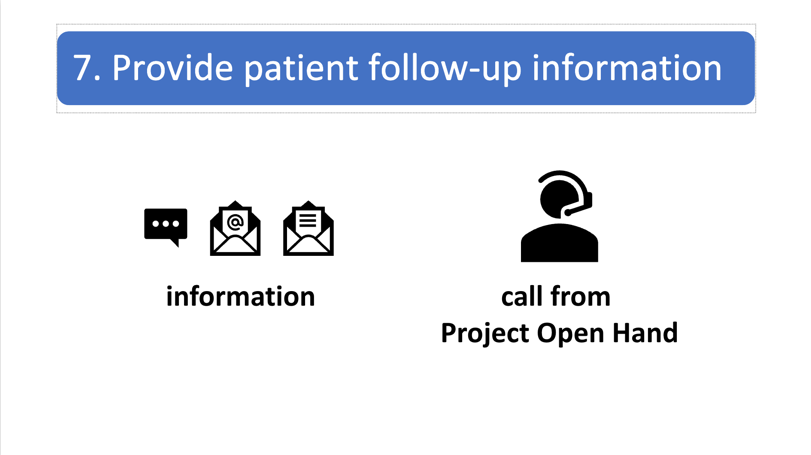


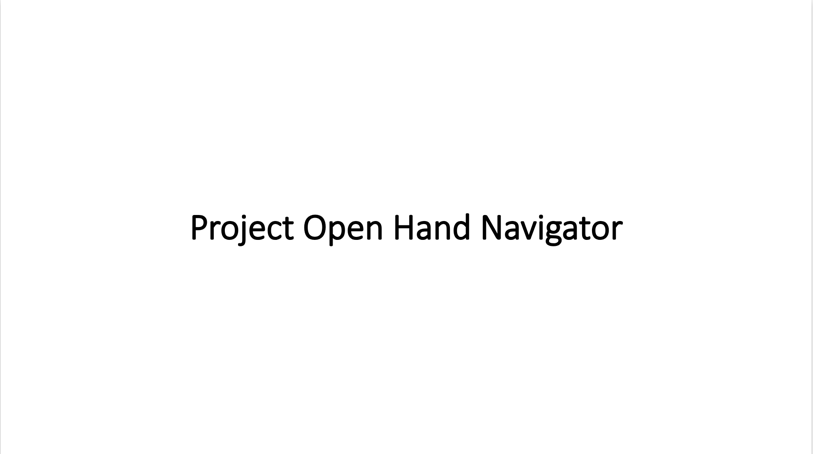


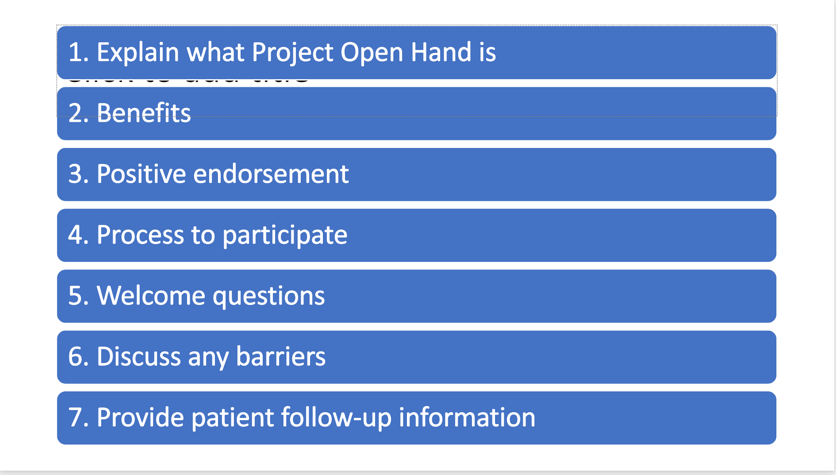


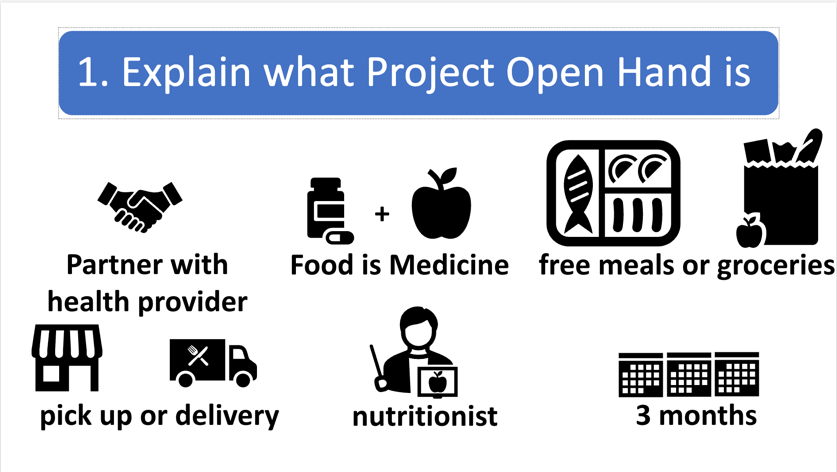

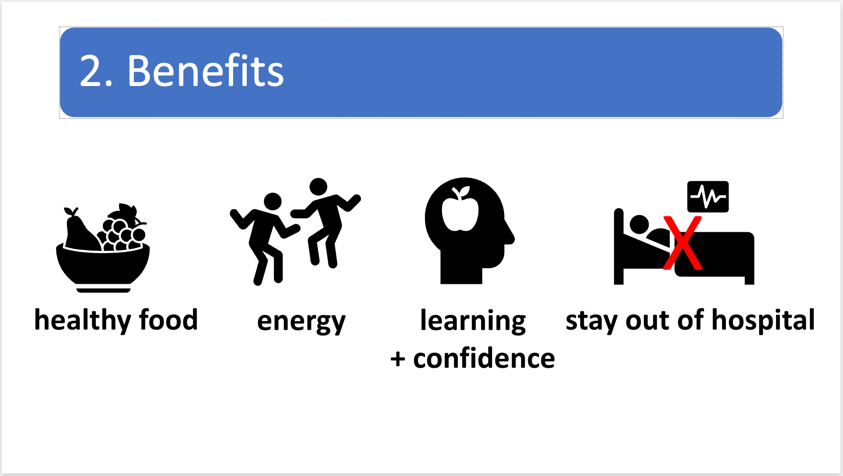

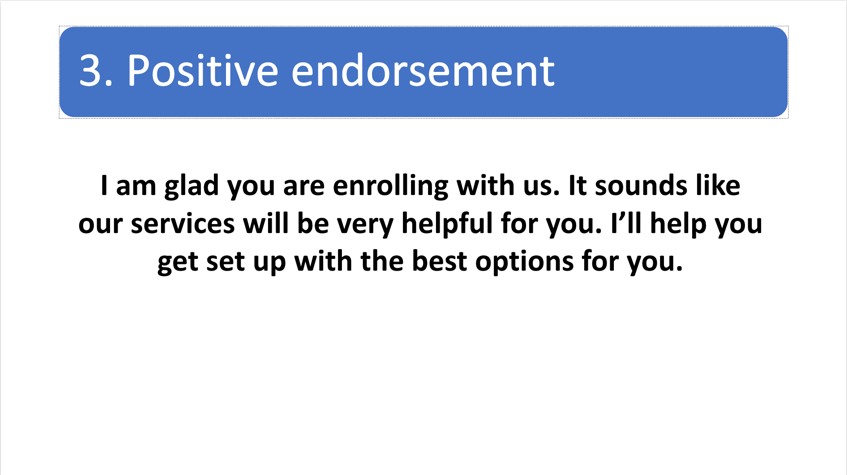

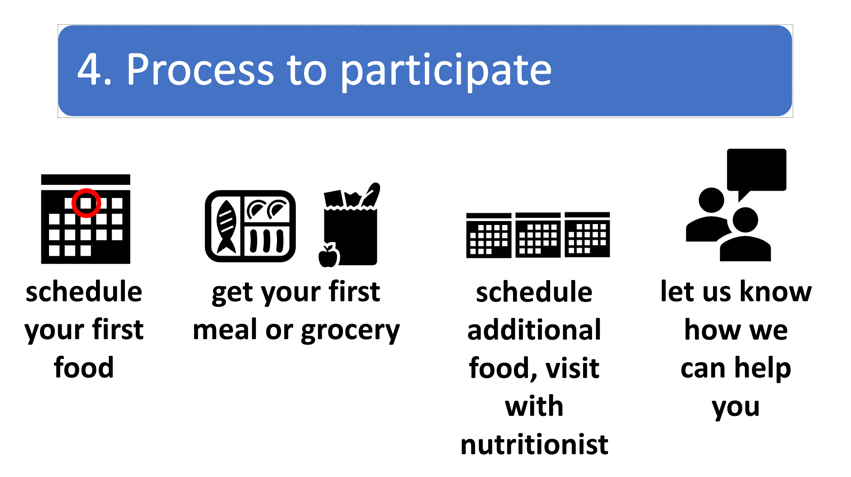


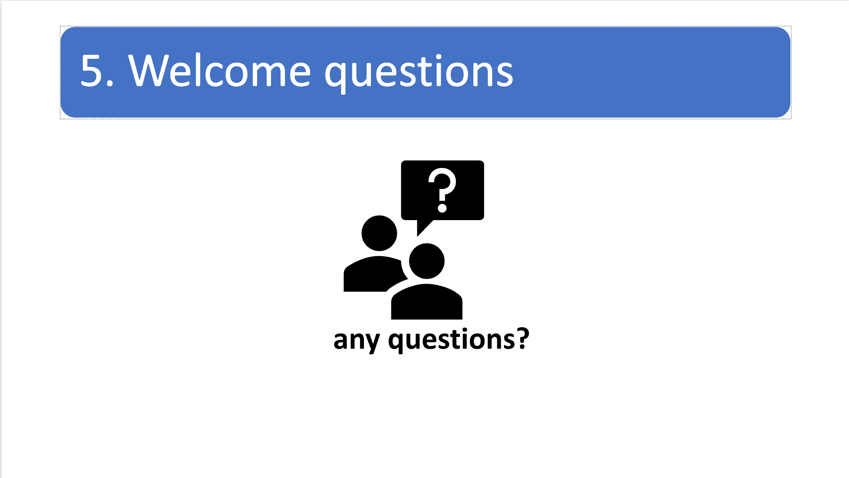

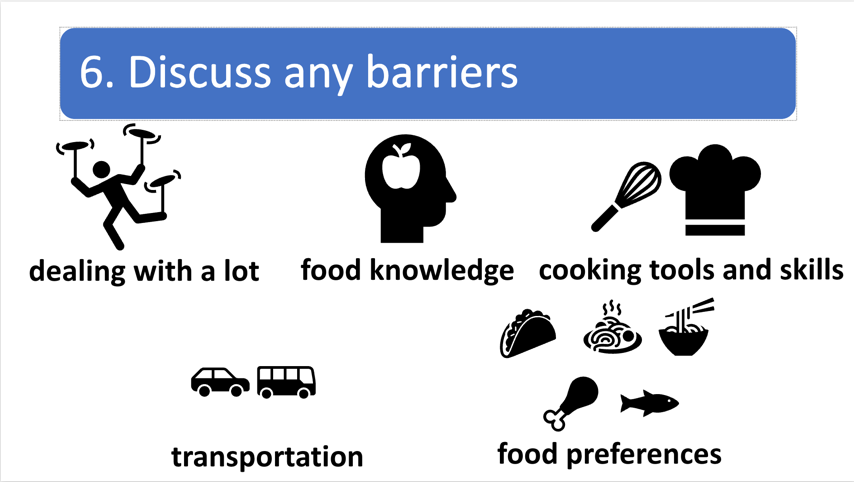

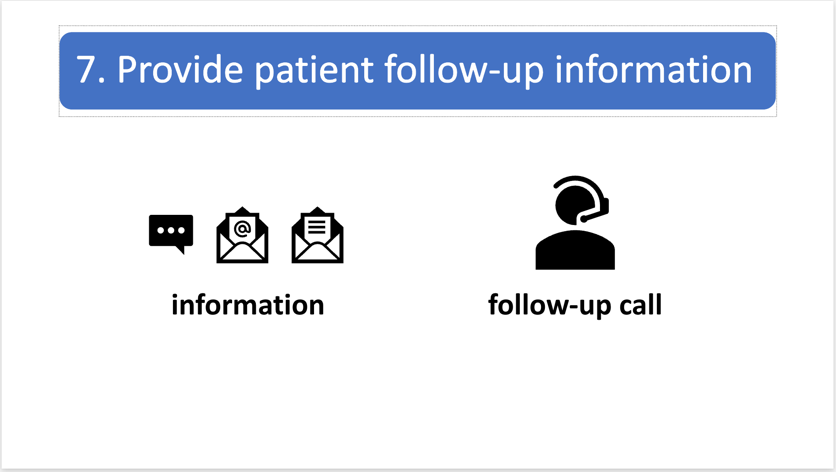

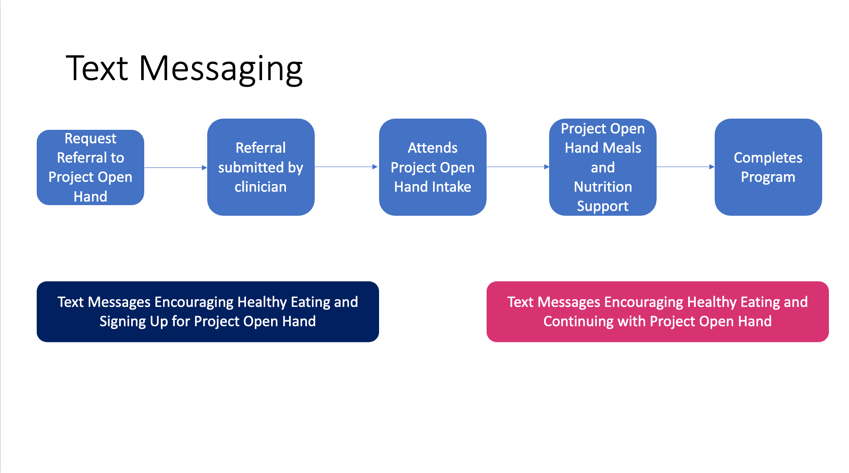

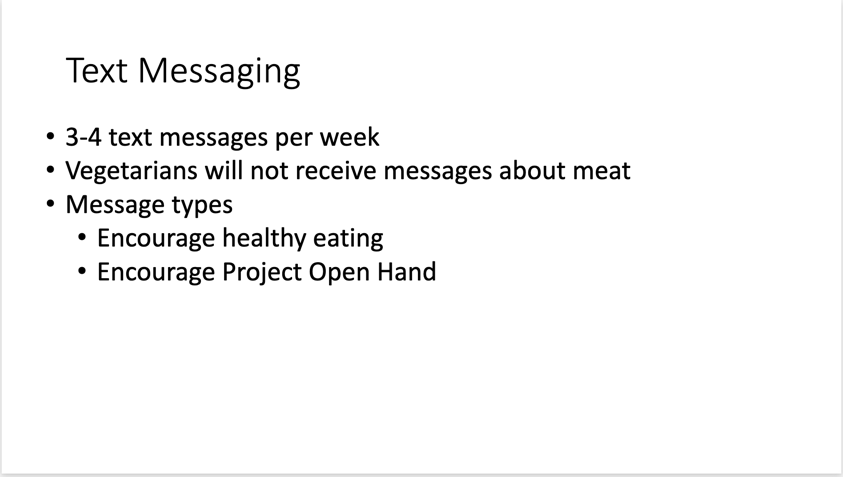

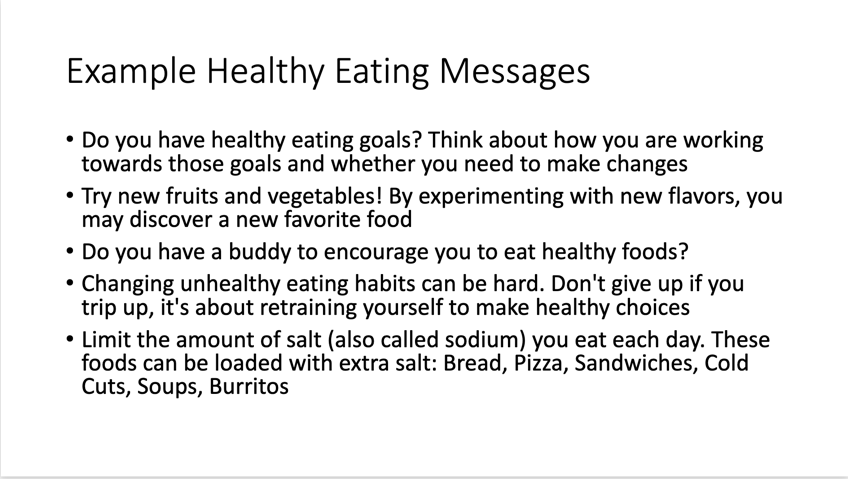


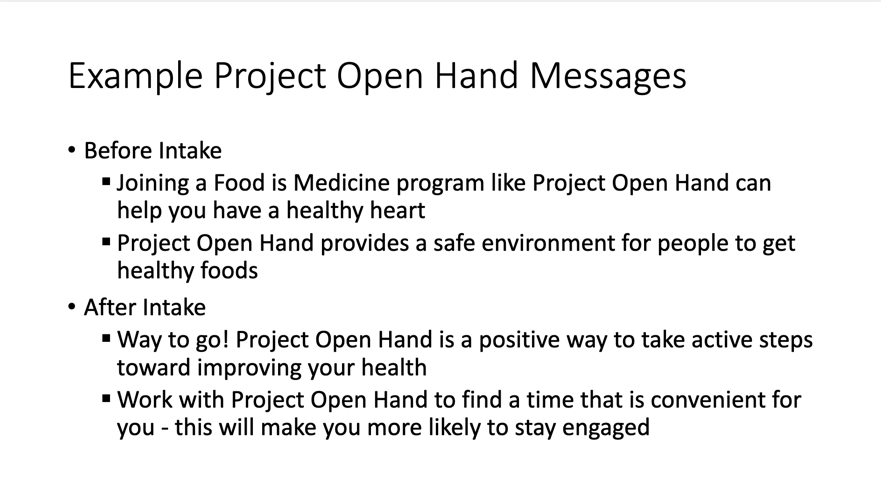

Supplement: Multimedia Appendix 2 [file formative-v10-e85650-s002.docx]
