# Supplementary material for: Optimizing Navigation and Text Messaging Interventions to Promote Participation in a Food Is Medicine Program Among People Participating in Cardiac Rehabilitation: Human-Centered Design Study
Source: JMIR Form Res. 2026 Apr 24;10:e85650. doi: 10.2196/85650 (PMC13122297; doi:10.2196/85650)
Supplement: Multimedia Appendix 3 [file formative-v10-e85650-s003.docx]

**Text Message Library**

| **Category** | **Text Message** | **Behavior Change Taxonomy** |
| --- | --- | --- |
| **Normal** | Welcome to the SUPeRFOOD Research Study! Eating healthy foods can help you have more energy and improve your heart health. | 5.1 Information about health consequences |
|  | Reading food labels can help you make healthier choices. Choose low sodium, low saturated fat, and low added sugars. Click to learn more! <https://bit.ly/4bJVmir> | 4.1 Instruction on how to perform the behavior |
|  | Avoid nighttime eating! If you are going to eat, choose a fruit or vegetable. Click to learn more! <https://bit.ly/45arvNJ> | 1.4 Action planning  12.3 Avoidance/reducing exposure to cues for the behavior |
|  | Changing unhealthy eating habits can be hard. Don't give up if you trip up, it's about retraining yourself to make healthy choices. | 1.2 Problem solving/coping planning  15.1 Verbal persuasion to boost self-efficacy |
|  | Savor the flavor! Eating too fast or when you're distracted may lead to consuming too many calories. Click to learn more! <https://bit.ly/3wZowei> | 5.2 Salience of consequences  5.5 Anticipated regret |
|  | Oatmeal, whole-grain cereals, and brown rice are some whole-grain foods that will help lower your cholesterol. Click to learn more! <https://bit.ly/3KvDkEB> | 4.1 Instruction on how to perform the behavior  5.1 Health consequences |
|  | Remove temptation by getting rid of all the convenient, unhealthy snacks. Click to learn what is considered an unhealthy snack. <https://bit.ly/3yVlKXR> | 12.1 Restructuring the physical environment |
|  | Create healthy habits and routines. If you reward yourself with sweets, try replacing it with a piece of fruit. Click to learn more! <https://bit.ly/3yDtRbt> | 8.3 Habit formation  8.2 Behavior substitution |
|  | Get through stressful times without eating: take a walk, talk to someone, do something you enjoy. | 1.2 Problem solving/coping planning  1.4 Action planning |
|  | Limit the amount of salt you eat. These foods can have extra salt: bread, cold cuts, soups, burritos. Click to learn more! <https://bit.ly/3yTjGj4> | 4.1 Instruction on how to perform the behavior  5.1 Information about health consequences |
|  | Make your plate colorful with vegetables such as yam, beets, or kale. What can you add to your plate to bring color? Click to learn more! <https://bit.ly/3Vr904c> | 1.4 Action planning  4.1 Instruction on how to perform the behavior |
|  | Looking for heart-healthy recipes? Click here for a few ideas! <https://recipes.heart.org/en/> | 4.1 Instruction on how to perform the behavior  7.1 Prompts/cues |
|  | The educational text messaging part of the study has finished. Thank you for participating! You will continue to receive text reminders to complete surveys. | N/A |
| **Extras: Normal** | Fruits and veggies are great sources of vitamins, minerals, and fiber. They can also help you control your hunger and weight. | 5.1 Information about health consequences |
|  | Heart-healthy eating means five servings of vegetables and fruit every day. Click to learn more! <https://bit.ly/4c1n4H6> | 1.1 Goal setting |
|  | To build a healthy eating style, limit sodas, processed foods, full-fat dairy, and tropical oils like coconut oil. Click to learn more! <https://bit.ly/3wZnmiU> | 4.1 Instruction on how to perform the behavior |
|  | If you're trying to eat healthier, write down what you are eating. You may find that extra calories have crept into your diet. | 2.3 Self-monitoring of behavior |
|  | The fats you eat can affect your cholesterol. Choose unsaturated fats like olive oil, avocado, or nuts more often. Click to learn more! <https://bit.ly/456UeCY> | 4.1 Instruction on how to perform the behavior  5.1 Information about health consequences |
|  | When eating out, order grilled foods instead of fried foods. | 4.1 Instruction on how to perform the behavior  8.2 Behavior substitution |
|  | When buying pre-cooked or processed food, look for the 'no salt' or 'low salt' option. Your heart will thank you. Click to learn more! <https://bit.ly/457fbOa> | 4.1 Instruction on how to perform the behavior |
|  | Pay attention to your feelings of hunger before, during and after eating. Use this awareness to recognize when you've had enough. | 6.2 Self-monitoring of outcome(s) of behavior |
|  | Find a friend who can support you in maintaining a healthy lifestyle. | 3.1 Social support (general) |
|  | Saturated fats from animal products can be bad your heart health. Choose low fat dairy instead of full fat. Click to learn more! <https://bit.ly/4e9tS7i> | 4.1 Instruction on how to perform the behavior  5.1 Information about health consequences  8.2 Behavior substitution |
|  | When cooking, use olive oil or canola oil instead of butter. Click to learn more! <https://bit.ly/4aLmwE3> | 4.1 Instruction on how to perform the behavior |
|  | Whole grains help control blood sugar and cholesterol and make you feel full. Click to learn more! <https://bit.ly/3RbgPZm> | 5.6 Information about emotional consequences  5.1 Information about health consequences |
| **Post** | Way to go! Project Open Hand is a positive way to take active steps toward improving your health. | 15.1 Verbal persuasion to boost self-efficacy  5.1 Information about health consequences |
|  | You can do it! Remind yourself that attending Project Open Hand is a great way to improve your heart health. | 15.1 Verbal persuasion to boost self-efficacy  5.1 Information about health consequences |
|  | Reward yourself for participating in Project Open Hand by doing something just for you - watch a movie! | 10.9 Self-reward |
|  | Who or what motivates you to eat healthy foods? Keep these motivations at the front of your mind to encourage yourself. | 8.1 Behavioral rehearsal/practice 15.4 Self-talk |
|  | Is your Project Open Hand plan working for you? Call the team at 415-447-2326 and let them know if you need to make a change. | 1.2 Problem solving/coping planning |
|  | Participating in Project Open Hand will make you healthier. Have you noticed a positive change? | 2.2 Feedback on behavior  5.1 Information about health consequences |
|  | Feeling stuck? Call Project Open Hand and ask to speak to a dietician 415-447-2326. | 3.1 Social support (general) |
|  | Write down 3 things that you like about healthy eating and put the list where you will see it often. | 1.4 Action planning  13.4 Self-affirmation |
|  | Try recreating your favorite foods from Project Open Hand for your friends and family! | 6.1 Demonstration of behavior/modeling  8.1 Behavioral practice/rehearsal |
|  | What are some heart-healthy recipes you can make with the groceries you get from Project Open Hand? | 1.1 Goal setting |
|  | Do you have feedback for Project Open Hand? Call 415-447-2326 to share your ideas. | 2.2 Feedback on behavior |
|  | Almost done with Project Open Hand? Create a plan to keep yourself going - start by writing down your favorite foods from Project Open Hand. | 1.4 Action planning |
| **Pre** | Project Open Hand can you help you achieve your healthy eating goals. Please look out for call from the Project Open Hand team to enroll you! | 1.1 Goal setting  3.1 Social support (general) |
|  | Feeling tired? Join Project Open Hand to eat heart-healthy foods and gain more energy! Call now at 415-447-2326! | 5.1 Information about health consequences  7.1 Prompts/cues  15.1 Verbal persuasion to boost self-efficacy |
|  | Are you still deciding to join Project Open Hand? Call 415-447-2326 if you have questions. | 7.1 Prompts/cues |
|  | Joining a "Food is Medicine" program like Project Open Hand can help you maintain a healthy heart. Call now at 415-447-2326! | 5.1 Information about health consequences  7.1 Prompts/cues |
|  | Make a plan and set some time aside for when you will sign up for Project Open Hand. Call now at 415-447-2326! | 1.4 Action planning |
|  | Meeting with a Project Open Hand dietician can improve your nutrition. | 3.1 Social support (general)  5.1 Information about health consequences |
|  | Overwhelmed as to where to start? Take the first step - call Project Open Hand at 415-447-2326. | 1.2 Problem solving/coping planning  7.1 Prompts/cues |
|  | Project Open Hand can be a great place to meet people who can give you support and encouragement. | 3.1 Social support (general) |
|  | Project Open Hand provides a safe environment for people to get heart-healthy foods. | 12.1 Restructuring the environment |
|  | Want to take a positive step toward a healthier life? Sign up for Project Open Hand today by calling 415-447-2326. | 13.3 Identity associated with changed behavior  15.1 Verbal persuasion to boost self-efficacy |
|  | Write 3 reasons you want to participate in Project Open Hand and keep the list where you will see it often. | 1.4 Action planning  2.3 Self-monitoring of behavior |
|  | Project Open Hand can give you the groceries you need to make heart-healthy meals. Call 415-447-2326 to join today! | 3.1 Social support (practical) |
