# Supplementary material for: Optimizing Navigation and Text Messaging Interventions to Promote Participation in a Food Is Medicine Program Among People Participating in Cardiac Rehabilitation: Human-Centered Design Study
Source: JMIR Form Res. 2026 Apr 24;10:e85650. doi: 10.2196/85650 (PMC13122297; doi:10.2196/85650)
Supplement: Multimedia Appendix 4 [file formative-v10-e85650-s004.docx]

**Navigator Scripts**

**Health System Navigator Script**

Hello Mr/Ms/XXXX,

**Intro**

This is *[your name]* with the SUPeRFOOD Research Study at UCSF. I’m calling for your weekly check-in. Is now still an ok time to talk? *[if not, reschedule]*

**Provide Encouragement**

I’m so glad that we are getting to talk today. It’s important for your heart health to learn about what’s good to eat.

**Today’s Visit**

Today we are going to talk more about signing up for Project Open Hand. We can also talk about whether there is anything that you need to help you eat healthier foods.

**Follow-up from Last Visit**

At our last visit, we talked about *[XXX]*.

*[If ready to sign-up]* Have you been able to have your intake with Project Open Hand?

- *[If yes]* That’s great! Are there any questions or concerns with getting started?
- *[If no but one is scheduled]* That’s great! Are there any questions or concerns with getting started?
- *[If no/Not Scheduled]* No problem. We can check in again next week.
- *[If still thinking about it]* Last time you mentioned *[XXX]*. What do you think about that now?

*If needed, can repeat steps from the initial visit. If steps from initial visit are not needed, skip to “Welcome Questions and Feedback”*

**Explain the Program**

Project Open Hand is a program that provides healthy meals and/or groceries to people with conditions, like heart problems. The Program will work with you to decide on getting meals or groceries. You can either go to their location in San Francisco, or they can arrange for the meals or groceries to be delivered to your home. You can also meet with a dietician to learn more about healthy food and how to choose the right foods for you.

**Explain the Benefits**

People who enroll in Project Open Hand can eat healthier foods, feel better, and in some cases prevent hospital stays.

**Provide Positive Endorsement**

You will benefit from joining Project Open Hand because *[provide individualized positive endorsement].*

**Describe Process to Enroll**

To enroll, you just have to let me know that you want to sign up. We will send an enrollment form to Project Open Hand for you. When you qualify, Project Open Hand will contact you about setting up an intake session.

**Welcome Questions and Feedback**

What questions do you have? Do you have any feedback for the program? We are learning from you!

**Address Barriers to Attendance**

*[use motivational interviewing techniques to assess likelihood and address barriers]*

On a scale from 0 to 10 (with 10 being most likely), how likely do you think you are to join Project Open Hand?

What led you to choose that number?

*[if other than 10]* What would it take for you to move to a higher number?

*Some barrier prompts:*

- *Is transportation a concern?*
- *Is scheduling or time a concern?*
- *Is communication or language a barrier?*
- *Do you have food preferences or restrictions?*
- *Do you have any questions about food preparation or storage?*

*[if patient is ready to sign-up, make sure referral has been completed, submitted, received]*

**Provide Follow-Up Information**

I will check in with you again next week to:

- *[if still considering]* Talk with you more about signing up for Project Open Hand
- *[if agrees to sign-up]* See if there are any questions or concerns with getting in for your first session with Project Open Hand.

When is a convenient time?

Would you like me to send follow-up information to your email, MyChart, paper mail, or a text message?

Thanks for taking the time to speak with me today!

**Food is Medicine Navigator Script**

**Intro**

This is *[your name]* with the SUPeRFOOD Research Study at Project Open Hand. I’m calling for your weekly check in. Is now still an ok time to talk? [if not, reschedule].

**Provide Encouragement**

I’m so glad that we are getting to talk today. It’s important for your heart health to learn about what’s good to eat.

**Today’s Visit**

Today we are going to talk more about Project Open Hand. I’d also like to learn more about you and whether there is anything that you need to help you learn about healthier foods.

**Project Open Hand Experience**

How has your experience with Project Open Hand been so far?

Any concerns?

Is there anything we can do to help you stay with the program?

*If needed, repeat initial elements. If initial elements not needed, skip to “Welcome Questions and Feedback”.*

**Explain the Benefits**

People who enroll in Project Open Hand can eat healthier foods, feel better, and in some cases prevent hospital stays.

**Provide Positive Endorsement**

We’re so glad that you are working with us at Project Open Hand.

**Describe Process to Participate**

We will set up a schedule for you to get meals/groceries and figure out how you will get the meals/groceries.

*[Provide individualized details about receiving groceries/meals.]*

**Welcome Questions and Feedback**

What questions do you have? Do you have any feedback for the program? We are learning from you!

**Address Barriers to Attendance**

*[use motivational interviewing techniques to assess likelihood and address barriers]*

On a scale from 0 to 10 (with 10 being most likely), how likely do you think you will be able to stay with Project Open Hand for 3 months?

What led you to choose that number?

*[if anything other than 10]* What would it take for you to move to a higher number?

*Some barrier prompts:*

- *Is transportation a concern?*
- *Is scheduling or time a concern?*
- *Is communication or language a barrier?*
- *Do you have food preferences or restrictions that differ from the available food options?*
- *Do you have any questions about food preparation or storage?*

**Provide Follow-Up Information**

I will check in with you again next week to see if there are any questions or concerns with working with Project Open Hand.

When is a convenient time?

Would you like me to send follow-up information to your email, paper mail, or a text message?

Thanks for taking the time to speak with me today!
