# Supplementary material for: Optimizing Navigation and Text Messaging Interventions to Promote Participation in a Food Is Medicine Program Among People Participating in Cardiac Rehabilitation: Human-Centered Design Study
Source: JMIR Form Res. 2026 Apr 24;10:e85650. doi: 10.2196/85650 (PMC13122297; doi:10.2196/85650)
Supplement: Multimedia Appendix 5 [file formative-v10-e85650-s005.docx]

**Participant Interview Guides**

# **Session 1**

**SUPeRFOOD Study**

**PARTICIPANT INTERVIEW GUIDE – SESSION 1**

**(Human Centered Design Initial Session)**

**Introduction**

Thank you for agreeing to talk with us today! We would like to ask you a few questions about your experience with healthy eating.

My name is <*insert name*>, and I am a team member for this project. I will be facilitating our discussion, which will take about 2 hours of your time. I am interested in all of your ideas, comments, and suggestions. There are no right or wrong answers. All comments, both positive and negative, are welcomed. Your opinion is important, and I want you to feel comfortable in saying what you really think. Because this is a group, we ask that you respect the other people in the group and keep what is said in the group private. This session will have two parts: a focus group discussion and feedback on a plan for a program to help people eat healthy foods. We will take a 5-minute break in between the two parts.

I want to make sure that we accurately capture all of your ideas; therefore, we would like to ask for your permission to record our discussion. However, we will keep this recording confidential and for use by people involved with the research only. Before we get started, I just want to remind you that your participation in this session is completely voluntary. You should feel free at anytime to let me know if you need a break or if you do not want to talk about a certain topic or answer a question. You can also let me know if you want to stop the session at any time.

Do you have any questions before we begin?

Before we begin, can each of you say, “I agree.”

I will now start the recording. *[Now start the recording.]*  *Start interview.*

Interview QUESTIONS (~30 minutes)

*Instructions for facilitator: These questions do not have to be read verbatim. They are topics to be used as a guide for steering the conversation. Generic prompts: If responses are limited or require clarification, probes may be used to elicit more detailed responses. Probes should use words or phrases presented by the participant using one of the following formats:*

*Tell me more about ________.*

*You mentioned ________; what did you mean by that?*

*Give me an example of _________?*

*Tell me about a time when _________?*

*Why do you think ________?*

1. What does a typical day of eating look like for you?
2. Tell me about the role that healthy eating currently plays in your life.
3. Who are the people in your life who play a role in what you eat?
4. Can you give an example of a time when you found it easy to eat healthy foods?
5. Can you give an example of time when you found it difficult to eat healthy foods?
6. How do you shop for food?
7. Project Open Hand is a program that helps people with health conditions get access to healthy meals or healthy groceries.
   1. Have you participated in Project Open Hand?
      1. If yes:
         1. How did you first hear about Project Open Hand?
         2. Tell me about the process to get started with Project Open Hand.
         3. Tell me about your experience with Project Open Hand.
         4. Probes: What made it easy or difficult?
      2. If no:
         1. How could Project Open Hand help you?
         2. What would keep you from joining Project Open Hand?
         3. What would keep you from staying with Project Open Hand?

*“Thanks for sharing many helpful ideas with us, and now we are coming near the end of our discussion, my last questions for you are…”*

1. Any other thoughts or ideas you would like to share?
2. What questions do you have?

*[10 minute break]*

Welcome back. I am going to show you something we call a User Journey. This describes how someone in cardiac rehabilitation could be referred to Project Open Hand and some ideas for things that could help someone participate in the project. As we go through the User Journey, we would like your feedback on journey and how we can make it work for people like you.

*[Go through slides on user journey]*

Any other thoughts or ideas you would like to share?

What questions do you have?

Thank you so much for your participation today.

## **Later Sessions**

**SUPeRFOOD Study**

**PARTICIPANT INTERVIEW GUIDE**

**(Human Centered Design Later Sessions)**

**Introduction**

Thank you for agreeing to talk with us today!

My name is <*insert name*>, and I am a team member for this project. I will be facilitating our discussion, which will take about 2 hours of your time. I am interested in all of your ideas, comments, and suggestions. There are no right or wrong answers. All comments, both positive and negative, are welcomed. Your opinion is important, and I want you to feel comfortable in saying what you really think. Because this is a group, we ask that you respect the other people in the group and keep what is said in the group private. This session will have two parts: [description of first part] and [description of second part]. We will take a 10 minute break in between the two parts.

I want to make sure that we accurately capture all of your ideas; therefore, we would like to ask for your permission to record our discussion. However, we will keep this recording confidential and for use by people involved with the research only. Before we get started, I just want to remind you that your participation in this session is completely voluntary. You should feel free at anytime to let me know if you need a break or if you do not want to talk about a certain topic or answer a question. You can also let me know if you want to stop the session at any time.

Do you have any questions before we begin?

Before we begin, can each of you say, “I agree.”

I will now start the recording. *[Now start the recording.]*

*Instructions for facilitator: Generic prompts: If responses are limited or require clarification, probes may be used to elicit more detailed responses. Probes should use words or phrases presented by the participant using one of the following formats:*

*Tell me more about ________.*

*You mentioned ________; what did you mean by that?*

*Give me an example of _________?*

*Tell me about a time when _________?*

Any other thoughts or ideas you would like to share?

What questions do you have?

Thank you so much for your participation today.
