# Supplementary material for: Optimizing Navigation and Text Messaging Interventions to Promote Participation in a Food Is Medicine Program Among People Participating in Cardiac Rehabilitation: Human-Centered Design Study
Source: JMIR Form Res. 2026 Apr 24;10:e85650. doi: 10.2196/85650 (PMC13122297; doi:10.2196/85650)
Supplement: Checklist 1 [file formative-v10-e85650-s006.docx]

**Consolidated criteria for reporting qualitative studies (COREQ): 32-item checklist**

| **No.** | **Item** | **Guide questions/description** | **Remarks** | **Page no.** |
| --- | --- | --- | --- | --- |
| **Domain 1: Research team and reflexivity** | | | | |
| Personal Characteristics | | | | |
| 1 | Interviewer/facilitator | Which author/s conducted the interview or focus group? | RDO, LC | 2 |
| 2 | Credentials | What were the researcher’s credentials? E.g. PhD, MD | Credentials are listed on the title page. | 1 |
| 3 | Occupation | What was their occupation at the time of the study? | Research coordinator (RDO)  Clinical research coordinator (LC) | N/A |
| 4 | Gender | Was the researcher male or female? | All researchers were female. | N/A |
| 5 | Experience and training | What experience of training did the researcher have? | All team members directly involved in the sessions had training in facilitating sessions and use of template analysis. | N/A |
| Relationship with participants | | | | |
| 6 | Relationship established | Was a relationship established prior to study commencement? | No prior relationship to the research participants was established prior to study commencement. | 2 |
| 7 | Participant knowledge of the interviewer | What did the participants know about the researcher? E.g. personal goals, reasons for doing the research | Participants did not report prior knowledge of the interviewer. | N/A |
| 8 | Interviewer characteristics | What characteristics were reported about the interviewer/facilitator? E.g. bias, assumptions, reasons and interests in the research topic | No characteristics were reported about the interviewer/facilitator. | N/A |
| **Domain 2: Study design** | | | | |
| Theoretical framework | | | | |
| 9 | Methodological orientation and Theory | What methodological orientation was stated to underpin the study? E.g. grounded theory, discourse analysis, ethnography, phenomenology, content analysis | Thematic analysis applied to pre-specified and emergent codes from the Theory of Planned Behavior. | 2-3 |
| Participant Selection | | | | |
| 10 | Sampling | How were participants selected? E.g. purposive, convenience, consecutive, snowball | Participants were selected via convenience sample. | 2 |
| 11 | Method of approach | How were participants approached? E.g. face-to-face, telephone, mail, email | Participants were approached via electronic health record (EHR) message. | 2 |
| 12 | Sample size | How many participants were in the study? | 6 people participated in the study. | 3 |
| 13 | Non-participation | How many people refused to participate or dropped out? Reasons? | We did not systematically collect data on refusal to participate. | N/A |
| Setting | | | | |
| 14 | Setting of data collection | Where was the data collected? E.g. home, clinic, workplace | Data was collected over Zoom meetings. | 2 |
| 15 | Presence of non-participation | Was anyone else present besides the participants and researchers? | Only participants and researchers were present in the human-centered design sessions. | 2 |
| 16 | Description of sample | What are the important characteristics of the sample? E.g. demographic data, date | The important characteristics of the sample were age and health literacy. | 2 |
| Data Collection | | | | |
| 17 | Interview guide | Were questions, prompts, guides provided by the authors? Was it pilot tested? | Interview scripts were prepared but were not pilot tested. | 3 |
| 18 | Repeat interviews | Were repeat interviews carried out? If yes, how many? | Repeat interviews were carried out for all sessions to accommodate conflicting schedules of the participants. | 2, 3 |
| 19 | Audio/visual recording | Did the research use audio or visual recording to collect the data? | All human-centered design sessions were audio recorded. | 3 |
| 20 | Field notes | Were field notes made during and/or after the interview or focus group? | Field notes were made during all sessions. | 3 |
| 21 | Duration | What was the duration of the interviews or focus group? | Each session lasted 90-120 minutes. | 2 |
| 22 | Data saturation | Was data saturation discussed? | Data saturation was not discussed. | N/A |
| 23 | Transcripts returned | Were transcripts returned to participants for comment and/or correction? | Transcripts were not returned to participants for comment and correction. | N/A |
| **Domain 3: Analysis and findings** | | | | |
| Data analysis | | | | |
| 24 | Number of data coders | How many data coders coded the data? | 3 individuals coded the data. | 3 |
| 25 | Description of the coding tree | Did authors provide a description of the coding tree? | There is no description of the coding tree. | N/A |
| 26 | Derivation of themes | Were themes identified in advance or derived from the data? | Themes were identified in advance. However, additional “emerging” themes were also derived from the data gathered. | 3-5 |
| 27 | Software | What software, if applicable, was used to manage the data? | REDCap was used to manage the data for all participants. | 2 |
| 28 | Participant checking | Did participants provide feedback on the findings? | Yes, participants provided feedback on our iterative intervention revisions through consecutive sessions and on the final intervention materials. | 3 |
| Reporting | | | | |
| 29 | Quotations presented | Were participate quotations presented to illustrate the themes/findings? Was each quotation identified? E.g. participant number | Participant quotations were provided to illustrate themes and findings. Quotations were not identified via participant number. | 4-7 |
| 30 | Data and findings consistent | Was there consistency between the data presented and the findings? | Yes, the findings were consistent with the data provided. | 4-8 |
| 31 | Clarity of major themes | Were major themes clearly presented in the findings? | Yes, major themes were presented in the results. | 4-7 |
| 32 | Clarity of minor themes | Is there a description of diverse cases or discussion of minor themes? | Yes, there is a discussion of minor themes in the results. | 4-7 |
